# Identification of Loliolide with Anti-Aging Properties from *Scenedesmus deserticola* JD052

**DOI:** 10.4014/jmb.2304.04044

**Published:** 2023-06-02

**Authors:** Dae-Hyun Cho, Jin-Ho Yun, Jina Heo, In-Kyoung Lee, Yong-Jae Lee, Seunghee Bae, Bong-Sik Yun, Hee-Sik Kim

**Affiliations:** 1 Cell Factory Research Center, KRIBB, Daejeon 34141, Republic of Korea; 2 Department of Integrative Biotechnology, Sungkyunkwan University, Suwon 16419, Republic of Korea; 3 Division of Biotechnology, Jeonbuk National University, Iksan 54596, Republic of Korea; 4 Department of Environmental Biotechnology, University of Science and Technology (UST), Daejeon 34113, Republic of Korea; 5 Korea Institute for Skin and Clinical Sciences, Konkuk University, Seoul 05029, Republic of Korea; 6 ASK LABS, KRIBB BVC Center 109, Daejeon 34141, Republic of Korea

**Keywords:** Anti-UV compound, fractionation, loliolide, microalgae, nuclear magnetic resonance spectroscopy (NMR), *Scenedesmus deserticola* JD052

## Abstract

Herein, different extracts of *Scenedesmus deserticola* JD052, a green microalga, were evaluated in vitro as a potential anti-aging bioagent. Although post-treatment of microalgal culture with either UV irradiation or high light illumination did not lead to a substantial difference in the effectiveness of microalgal extracts as a potential anti-UV agent, the results indicated the presence of a highly potent compound in ethyl acetate extract with more than 20% increase in the cellular viability of normal human dermal fibroblasts (nHDFs) compared with the negative control amended with DMSO. The subsequent fractionation of the ethyl acetate extract led to two bioactive fractions with high anti-UV property; one of the fractions was further separated down to a single compound. While electrospray ionization mass spectrometry (ESI-MS) and nuclear magnetic resonance (NMR) spectroscopy analysis identified this single compound as loliolide, its identification has been rarely reported in microalgae previously, prompting thorough systematic investigations into this novel compound for the nascent microalgal industry.

## Introduction

While skin aging is known to occur through either intrinsic or abrupt extrinsic processes [1], extrinsic aging in particular is known to be accelerated by harmful environmental factors, with chronic ultraviolet (UV) exposure being identified as a major culprit [2]. Indeed, UV irradiation is closely linked to atrophy, pigmentation, wrinkles, and even skin cancer [3], and its penetration could cause intracellular damage in various types of skin cells, including keratinocytes and dermal fibroblasts. Previous studies have established that excessive UV irradiation induces the production of reactive oxygen species (ROS), cytokine synthesis, and pyrimidine dimer formation [4], each of which could activate the mitogen-activated protein kinase (MAPK) signaling pathway and thus increase the expression of senescence marker proteins p21 and p16 as well as the expression of matrix metalloproteinases (MMP) in dermal fibroblasts [5]. Considering “no question” on the causal link between excessive UV exposure and skin aging, identifying an effective UV protection agent has been a priority of researchers and practitioners in the field of cosmetic science with a focus on identifying new functional ingredients that could either directly protect against UV irradiation or mitigate UV-damage associated aging mechanisms [6].

Microalgae are photosynthetic microorganisms and are considered to be some of the oldest creatures on the planet [7]. In particular, they produce a variety of secondary metabolites that enable their survival in a wide range of habitats: many strains of microalgae are well identified to proliferate even under prolonged exposure to environmental stressors, including high UV irradiation, and previous studies elucidated a critical role of secondary metabolites, including pigments and fatty acids, on algal adaptation to hostile environmental conditions [8]. Not surprisingly, these metabolites are known to offer high nutraceutical value with a number of potential application routes being actively explored [8]. For instance, studies have demonstrated that chlorophylls, carotenoids, and various pigment derivatives can be used as food and cosmetic ingredients [9].

Belonging to Chlorophyta [10], *Scenedesmus deserticola* is a strain of microalgae that has been previously reported to survive in dry as well as strong UV exposure conditions [11], suggesting the presence of functional biocompound(s) that could act as an effective UV protection agent. Herein, ethyl acetate extract from the biomass of *Scenedesmus deserticola* JD052 treated under different stress conditions (*i.e.*, high light and UV), previously discussed as inducing the accumulation of bioactive compounds [8], was explored as a potential functional ingredient with anti-UV property. While the results indicated the effectiveness of the extract as an optical barrier and in improving damaged skin, the ethyl acetate extract was fractionated using thin-layer chromatography (TLC) and high-performance liquid chromatography (HPLC). Moreover, damage protection test against UV radiation was systematically performed to identify an effective single substance; nuclear magnetic resonance (NMR) analysis then determined the structure of the active fraction as loliolide, a compound previously reported to present in plants and seaweeds with antibacterial, anticancer, skin moisturizing, hair-growth promoting, and antioxidant properties [12-14].

## Materials and Methods

### Strain Cultivation and Extraction

*Scenedesmus deserticola* JD052 was cultured in BG11 medium in a 5 L photo-bioreactor (Sigma Aldrich, USA) for 7 days at 25°C, under continuous light (150 μmol m^-2^ s^-1^) [15] and an equivolume of the cellular culture was then divided into three photobioreactors. While one of three PBRs was maintained under the identical light condition as the preceding cultivation period, two reactors were subjected to post-treatment either under high light intensity (1,200 μmol m^-2^ s^-1^) or UVB radiation (30 μmol m^-2^ s^-1^) for 12 h to elucidate the influence of each post-treatment on the accumulation of anti-UV compounds. Microalgal cells were then collected and lyophilized; extraction of lyophilized biomass samples collected under different cultivation conditions (*i.e.*, samples without post-treatment, samples post-treated with UV irradiation, samples post-treated with high light intensity) were disrupted by ultrasonication for 10 min after adding 150 – 160 mg of lyophilized biomass in one of four solvents (*i.e.*, ethanol, ethyl acetate, hexane, and distilled water). Each extract was then filtered through 0.45 μm membrane filter and the solvent was removed using a rotary evaporator (Buchi, Switzerland), which was subsequently dissolved in DMSO for comparative in vitro assays [2]. Large scale culture for fractionation and NMR analysis was performed using BG11 medium in 5,000 L cylindrical photobioreactor; 1.2 kg of the harvested and lyophilized sample was then added in 80 L of ethyl acetate, which was subjected to ultrasonication for 3 hr as well as homogenization at 50°C for another 3 hr. While the resulting extracts were filtered through a PVDF filter and distilled before further use, all distilled extracts were stored at -20°C.

### Human Skin Cell Cultivation

Normal human dermal fibroblasts (nHDFs) were purchased from Lonza (Basel, Switzerland) and grown in Dulbeccós modified Eaglés medium (DMEM) (Gibco Life Technologies, USA) supplemented with 10% fetal bovine serum (FBS) (Thermo Fisher Scientific, USA). Cells were maintained in a humidified atmosphere with 5%CO_2_ at 37°C.

### Human Skin Cell Viability Assay

Water-soluble tetrazolium salt-1 (WST-1) assay (EZ-Cytox Cell Viability Asay Kit, ITSBIO, Korea) was performed to evaluate the influence of microalgal extract on the viability of human skin cell [3]. nHDF (4 × 10^3^) cells were first seeded, cultured, and treated for 24 h with extracts at the concentration of 0, 1, 5, 10, or 20 μg/ml. 1/ 10 culture volume of the WST-1 analysis solution was added to each well and cultured for 0.5 h. Cell survival was determined using a 620 nm reference filter and an iMark plate reader (Bio-Rad, USA) at 450 nm. All viability measures were presented as the mean ± standard deviation (SD) based on three independent replicates.

### Exposure of nHDFs to UVB Radiation

UV lighting machine Super Light VI (Boteck, Korea) was deployed as a source of UVB irradiation for the exposure test. nHDF (2 × 10^5^ cells/plates) was seeded onto 60 mm cell culture plates and incubated for 24 h; nHDFs amended with microalgal extract either before or after the exposure to UVB were compared as well as nHDFs treated with the corresponding extract both before and after UVB exposure. For pre-exposure treatment group, HDFs were treated with microalgal extract for 6 h, after which the medium was washed with phosphate-buffered saline (PBS). Subsequently, 20 mJ/cm^2^ of UVB was applied to the culture plate after removing the lid. While PBS was replaced with fresh growth medium and cells were incubated for additional 24 h, post-exposure treatment groups were treated with the extract added medium for 24 h.

### Fractionation of Active Biocompound

Approximately 250 g of the ethyl acetate extract with confirmed efficacy was dissolved in chloroform and was subjected to silica gel column chromatography analysis. As the elution solvent, chloroform and chloroform-methanol were sequentially used and the volumetric ratio of chloroform and methanol was changed from 100:1 (v/v) to 1:1 (v/v); all fractions were attained by silica gel TLC, and each fraction was analyzed by HPLC. In addition, preparative Octadecylsilyl medium-pressure liquid chromatography (ODS-MPLC) (CombiFlash RF+, Teledyne ISCO, USA) was performed to confirm the fractioned compound [16].

### Analytical Methods

Electrospray ionization (ESI)-mass was performed using ESI-QTRAP-3200 mass spectrometer (Applied Biosystems, USA). In addition, nuclear magnetic resonance (NMR) spectra were obtained using JEOL JNM-ECZ500R, 500 MHz FT-NMR Spectrometer at 500 MHz for ^1^H NMR and at 125 MHz for ^13^C NMR in CD3OD (Jeol, Japan). Chemical shifts were given in ppm (δ) relative to tetramethylsilane. For NMR spectra, two-dimensional NMR such as ^1^H-^1^H correlated spectroscopy (COSY), heteronuclear single quantum coherence spectroscopy (HMQC), and heteronuclear multiple bond correlation (HMBC) as well as one-dimensional NMR such as ^1^H NMR and ^13^C NMR were employed [17, 18].

### Statistical Analysis

Student’s two-tailed t-test was performed on replicated data with a significance level of 0.05, and the results were presented as either mean values or mean values ± standard deviation.

## Results

### Microalgal Cultivation and Extraction

Following the cultivation of *S. deserticola* JD052 for 7 days at a working volume of 3 L in a 5 L photo-bioreactor, the culture volume was equally divided into 3 portions in separate reactors to treat them under different post-treatment conditions for 12 h: (1) normal light condition (150 μmol m^-2^ s^-1^) (2) high light condition (1,200 μmol m^-2^ s^-1^ )(3) UV irradiation (30 μmol m^-2^ s^-1^). Upon the cultivation and 12-h long post-treatment, the cellular density of 2.01 g/l, 1.90 g/l, and 1.74 g/l were respectively observed in the cultures subjected to the post-treatment with normal light (*i.e.*, negative control), high light intensity, and UV irradiation. Each biomass sample was then extracted using 4 different solvents (*i.e.*, ethanol, ethyl acetate, hexane, and distilled water) to test its potential as a UV protection agent. A total of 12 extracts were obtained and 55 g of ethyl acetate extract was obtained additionally from 1.2 kg of dry biomass that was produced in a 5,000 L photobioreactor for stepwise fractionation and NMR.

### Evaluation of Cytotoxicity of *S. deserticola* Extracts

**Fig. 1:**
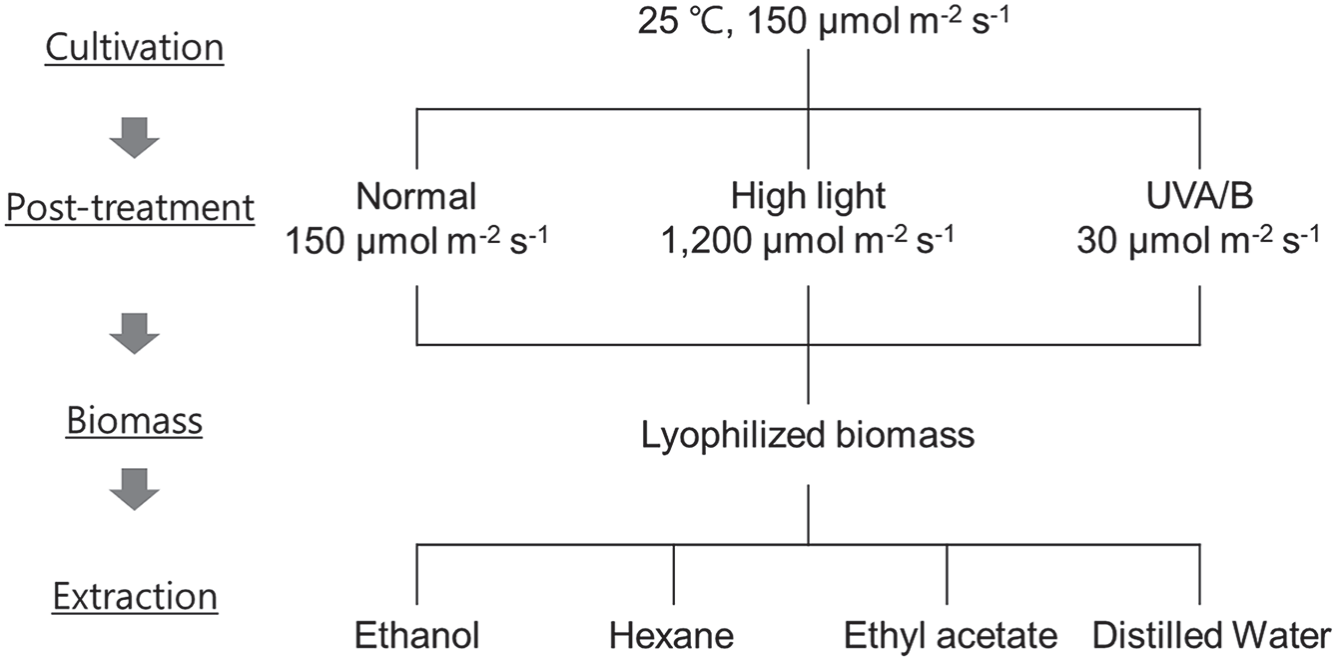
Schematic summary of extract preparation from *S. deserticola*. The alga was cultured and exposed to different stress conditions: normal light, high light, and UV radiation. The algal extracts were then prepared using different solvents (*i.e.*, ethanol, ethyl acetate, hexane, and distilled water) following sonication.

**Fig. 2:**
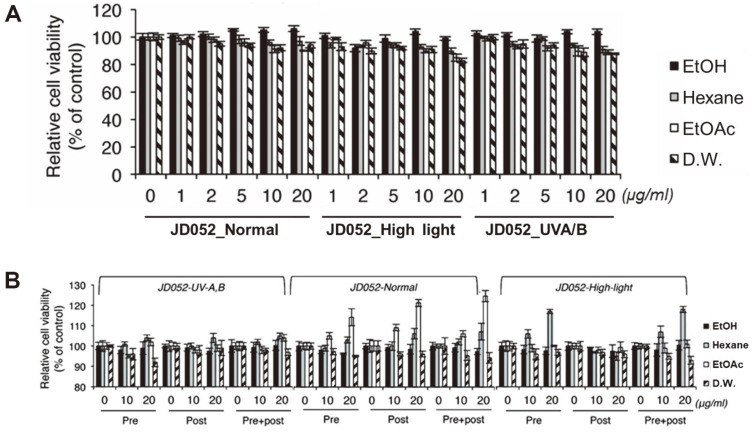
Cytotoxicity and photoprotective effect of *S. deserticola* JD052 extracts on UVB-induced cytotoxicity in nHDFs. (**A**) Cellular viability upon the treatment of different concentrations of microalgal extract with no UVB exposure (**B**) cell viability obtained with nHDFs treated with the corresponding microalgal extract with UVB exposure. For (**B**), Microalgal extracts were treated were treated before or after UV exposure (see text). nHDF cells were exposed to UV stress by exposing them to 20 mJ/cm^2^ UVB. Each value is the mean ± SD from three independent experiments. Asterisk (*) denotes *p* < 0.01, compared with the control group treated with DMSO.

After preparing 12 extracts from *S. deserticola* (Fig. 1), their cytotoxicity on nHDFs was evaluated by WST-1-based cell viability assay using DMSO as the control. As shown in Fig. 2, the extracts obtained with ethanol, hexane, and ethyl acetate had a negligible cytotoxic effect on nHDFs with less than 10% decrease in cell viability. However, DW-derived extracts indicated a higher cytotoxicity than the rest of the extracts.

### Effect of *S. deserticola* Extracts on UVB-Induced nHDF Cytotoxicity

The effectiveness of the extracts of *S. deserticola* was evaluated based on the influence of different extracts on UVB-induced nHDF cytotoxicity. To this end, nHDFs were divided into pretreatment (group 1), post-treatment (group 2), and pre- and post-treatment (group 3) groups. Specifically, pretreatment group was treated with the corresponding extract for 6 hr prior to the exposure to UVB radiation; post-treatment group was treated with the extract for 24 h after UVB exposure; and pre- and post-treatment group was amended with the corresponding extract before and after UVB exposure for 6 h and 24 h, respectively. In all test conditions, UVB irradiation of 20 mJ/cm^2^ was applied and the cytotoxicity of each group was analyzed using WST-1 assay.

The results indicated no UVB resistance effect in the extracts from microalgal biomass post-treated with UVA/B (Fig. 2B). Notably, the hexane extract of the biomass sample exposed to high light intensity indicated 17%increase in its UVB resistance effect (*i.e.*, greater nHDFs viability) after the 6-hr pretreatment 6-hr pretreatment, compared with DMSO-treated negative controls (Fig. 2B). Among the extracts obtained from the normal light condition, the ethyl acetate extract exhibited protective activity against UVB cytotoxicity, with a maximum cellular viability observed in the conditions treated with 20 μg/ml of the ethyl acetate extract, which corresponded to 15% increase in cellular viability compared with that of the control (Fig. 2B). Interestingly, UVB resistance was also observed in the post-treatment group: post-treatment with 20 μg/ml of ethyl acetate extract from the biomass sample not amended with either high light or UV irradiation treatment led to 21% increase in its viability compared to the DMSO-treated control. While the maximum UVB resistance effect was observed in the group 3 with 25% increase in cellular viability, the results collectively suggested the effectiveness of these algal extracts in remediating UV-induced damage with or without the post-treatment of microalgal culture.

### Fractionation and Screening

Upon confirming the effectiveness of ethyl acetate extract on increasing the viability of nHDFs, identification of an active biocompound was further performed using biomass sample obtained from the large-scale production of *S. deserticola*. In total, 55 g of ethyl acetate extract was obtained for the fractionation of an active biocompound following the production of 1.2 kg of dry biomass in 5,000 L bioreactor. An active fraction was concentrated under reduced pressure and re-chromatographed on a column of silica gel eluted with chloroform: methanol (50:1 → 1:1, v/v). Thereafter, active fractions were combined, concentrated, and chromatographed on a Sephadex LH-20 column eluted with 70% aq. methanol. An active fraction was concentrated and separated by preparative reversed-phase (ODS) MPLC using a gradient elution from 10% aq. methanol to 100% methanol. Finally, an active fraction was separated by preparative HPLC (Cosmosil Rp-18 column, f 10 × 150 mm, flow rate of 3 mL/min) eluted with 30% aq. methanol.

**Fig. 3:**
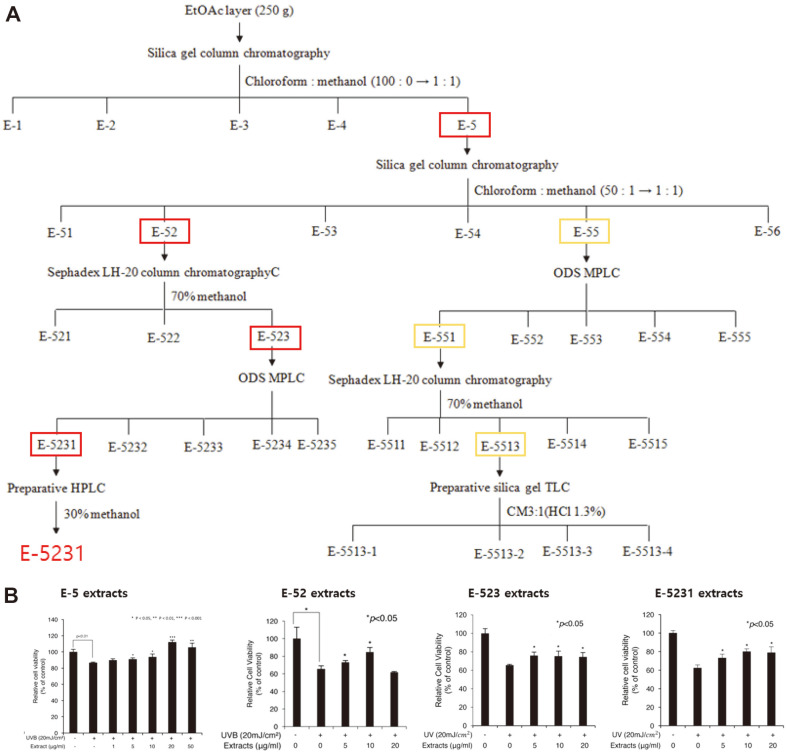
The fractionation and screening from the *S. deserticola* JD052 extract. (**A**) Schematic of the isolation and purification (**B**) Evaluation of the cellular viability against UVB irradiation for selected fractions.

While ethyl acetate extract was separated into 5 fractions (*i.e.*, E-1 to E-5), anti-UV activity was observed with 20 μg/ml of E-5, confirming the existence of active compound in this fraction. E-5 fraction was further separated into 6 fractions (E-51 to E-56); UV-protection effect was observed in E-52 and E-55; and additional separation was performed with E-52 and E-55. Although no single substance with efficacy was found in E-55, E-52 fraction was additionally separated and a single effective substance was identified and labeled as E-5231 (Fig. 3).

### Structure Determination

**Fig. 4:**
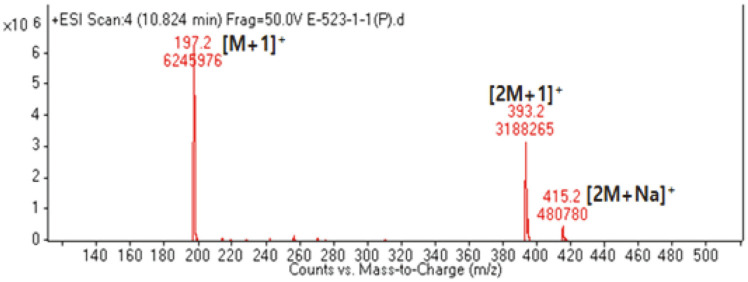
Analysis of molecular weight using electrospray ionization mass (ESI-MS).

**Fig. 6:**
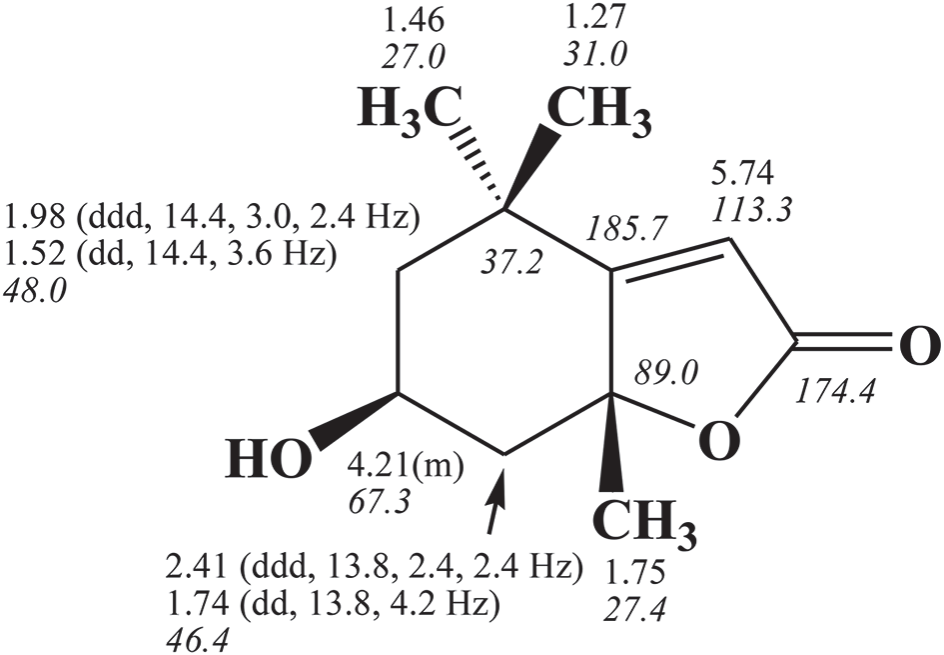
^1^H- and ^13^C (italics)-NMR chemical shifts of Fig. 5.

E-5231 fraction exhibited quasi-molecular ion peaks at *m/z* 197.2 [M+H]^+^, 393.2 [2M+H]^+^, and 415.2 [2M+Na]^+^ in the electrospray ionization mass (ESI-MS), suggesting a molecular weight of 196 (Fig. 4). The ^1^H NMR spectrum of E-5231 showed signals due to one olefinic methine at δ 5.74, one oxygenated methine at δ 4.21, two inequivalent methylene groups at δ 2.41/1.74 and 1.98/1.52, and three singlet methyl groups at δ 1.75, 1.46, and 1.27. In the ^13^C NMR spectrum, eleven carbon peaks including one ester carbonyl carbon at δ 174.4, one sp2 quaternary carbon at δ 185.7, one olefinic methine carbon at d 113.3, one oxygenated quaternary carbon at δ 89.0, one oxygenated methine carbon at δ 67.3, two methylene carbons at δ 48.0 and 46.4, one quaternary carbon at δ 37.2, and three methyl carbons at δ 31.0, 27.4, and 27.0 were evident. All proton-bearing carbons were verified by heteronuclear multiple quantum coherence (HMQC) spectrum (Fig. S9), and the ^1^H-^1^H correlation spectroscopy (^1^H-^1^H COSY) spectrum revealed a partial structure, -CH_2_-CH(-O)-CH_2_- (Figs. S6-S8). The structure of E-5231 was determined by heteronuclear multiple-bond coherence (HMBC) spectrum (Fig. S10), which showed long-range correlations from two methyl protons at δ 1.46 and 1.27 to the carbons at d 185.7, 48.0, and 37.2, from the methylene protons at δ 2.41/1.74 to the carbons at d 185.7 and 48.0, and from the methyl protons at δ 1.75 to the carbons at d 185.7, 89.0, and 46.4 (Fig. 6). Finally, long-range correlations from the olefinic methine proton at δ 5.74 to the carbons at d 185.7, 174.4, 89.0, and 37.2, and the elimination process established the structure of E-5231 as shown in Fig. 6. Combined together, compound E-5231 was identified as loliolide and the NMR spectral data of E-5231 matched well with those available in previous peer-reviewed reports [17, 18].

## Discussion

UV-ray is the main cause of various skin diseases and aging by inducing ROS production and pyrimidine dimer formation, which are directly associated with cell aging and death [19, 20]. Therefore, understanding the mechanism of skin damage caused by UV-rays and establishing measures to mitigate them has become increasingly important [19]. Recent studies have shown that microalgae extracts can effectively block UV-rays and repair damaged skin; this UV-protection effect is partially attributed to the presence of photosynthetic pigments and other secondary metabolites [2, 3].

This study investigated the effects of different light treatments amended after the initial cultivation period of *S. deserticola* JD052. Interestingly, UVA/B irradiation as a post-treatment did not lead to a greater UV protection activity of microalgal extracts in vitro regardless of the type of solvent used. However, microalgal culture post-treated with high light intensity seemingly resulted in an increased UV protection activity in the case of the hexane extract. Furthermore, microalgal culture not post-treated with light stress conditions also exhibited UV protective activity; the protective activity was observed in both nHDFs treated with ethyl acetate extract before or after UVB irradiation, suggesting a high likelihood of the existence of UV protection compound in the ethyl acetate extract in *S. deserticola* cultured in normal cultivation conditions.

While identifying a bioactive compound present in the extract necessitated a large quantity of biomass sample for stepwise fractionation, 5,000-L cylindrical photobioreactor was operated and biochemical fractions exhibiting UV protective activity in vitro was identified. Notably, although one of the fractions, E-5231, clearly exhibited the desired activity, the fraction E-55 could not be fractioned down to a single compound even with a confirmed UV protection activity. Thus, it was likely that UV protective activity observed with the ethyl acetate extract of *S. deserticola* could be mediated by a mixture of active compounds, in addition to the identified single compound [21].

The subsequent NMR and electrospray ionization mass (ESI-MS) performed on the single fraction E-5231 enabled the determination of the molecular weight and structure of this unknown compound of interest: this single active compound has a molecular weight of 196.24 MW and its structure clearly pointed it as loliolide, a monoterpenoid lactone. Loliolide has been previously reported to exhibit antibacterial, anticancer, skin moisturizing, and antioxidant activities [12-14], while these reports were mostly limited to plants and seaweeds.

Considering few studies available on microalgae-derived loliolide, the analytical results present herein is, to our knowledge, the first report detailing the structure of microalgae-derived loliolide – Importantly, this novel compound could be produced in conventional or newly designed production systems in a sustainable manner by utilizing atmospheric CO_2_ and/or excessive nutrients present in different wastewater sources. Given that no additional post-treatment may be necessary, the overall design and operation of *S. deserticola* cultivation could be simplified, even though further investigations into the influence of different cultivation conditions (*e.g.*, temperature, pH, salinity) could further identify effective conditions that could induce high accumulation of the identified active compound. In addition, while the effectiveness of different microalgal extracts were evaluated only with nHDF cells, more systematic investigations will be necessary, especially with a focus on achieving in-depth understanding of molecular mechanism(s) behind the UV protective property of loliolide as well as testing the effectiveness of loliolide and different fractions on other types of human skin cells, which could open new commercialization opportunities for this autochthonous microalga.

## Supplemental Materials

Supplementary data for this paper are available on-line only at http://jmb.or.kr.

## Figures and Tables

**Fig. 5:**
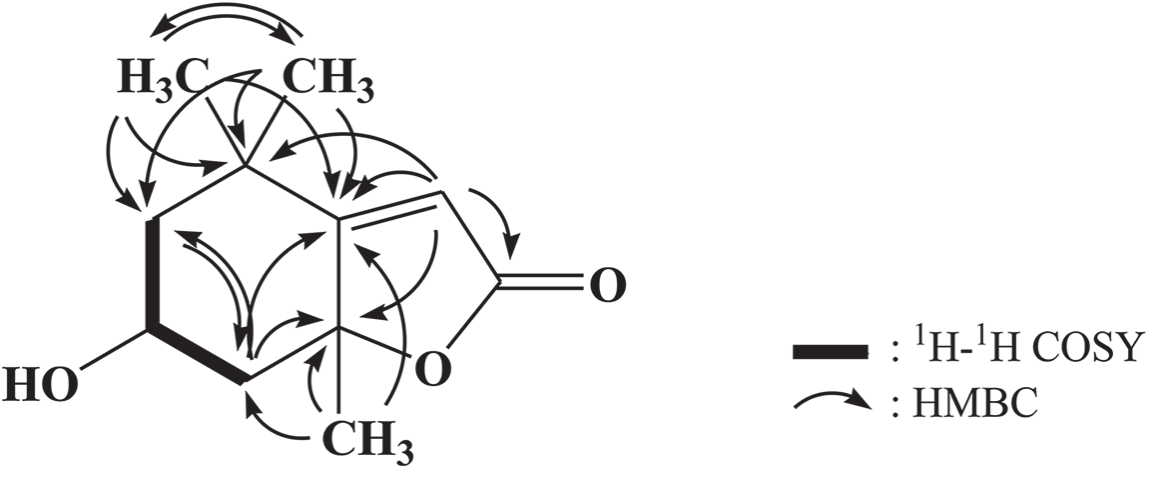
Two-dimensional NMR correlations.

## References

[1] Resende DISP, Ferreira M, Magalhães C, Sousa Lobo JM, Sousa E, Almeida IF (2021). Trends in the use of marine ingredients in antiaging cosmetics. Algal. Res..

[2] Lee JJ, Kim KB, Heo J, Cho DH, Kim HS, Han SH (2017). Protective effect of *Arthrospira platensis* extracts against ultraviolet Binduced cellular senescence through inhibition of DNA damage and matrix metalloproteinase-1 expression in human dermal fibroblasts. J. Photochem. Photobiol. B Biol..

[3] Lee JJ, An S, Kim KB, Heo J, Cho DH, Oh HM (2016). Extract of *Ettlia* sp. YC001 exerts photoprotective effects against UVB irradiation in normal human dermal fibroblasts. J. Microbiol. Biotechnol..

[4] Banaś AK, Zgłobicki P, Kowalska E, Bażant A, Dziga D, Strzałka W (2020). All you need is light. Photorepair of uv-induced pyrimidine dimers. Genes (Basel).

[5] Kim H-M, Jung JH, Kim JY, Heo J, Cho D-H, Kim H-S (2018). The protective effect of violaxanthin from *Nannochloropsis oceanica* against ultraviolet B-induced damage in normal human dermal fibroblasts. Photochem. Photobiol..

[6] Salminen A, Kaarniranta K, Kauppinen A (2022). Photoaging: UV radiation-induced inflammation and immunosuppression accelerate the aging process in the skin. Inflamm. Res..

[7] Murad ME, Al-Dawody M (2020). Biodiesel production form spirulina microalgae and its impact on diesel engine characteristicsreview. Al-Qadisiyah J. Eng. Sci..

[8] Mutschlechner M, Walter A, Colleselli L, Griesbeck C, Schöbel H (2022). Enhancing carotenogenesis in terrestrial microalgae by UVA light stress. J. Appl. Phycol..

[9] Kim U, Cho D-H, Heo J, Yun J-H, Choi D-Y, Cho K (2020). Two-stage cultivation strategy for the improvement of pigment productivity from high-density heterotrophic algal cultures. Bioresour. Technol..

[10] Lewis LA, Flechtner VR (2004). Cryptic species of *Scenedesmus* (Chlorophyta) from desert soil communities of Western North America. J. Phycol..

[11] Terlova EF, Lewis LA (2019). Tetradesmus bajacalifornicus L.A.Lewis & *Flechtner*, sp. nov. and Tetradesmus deserticolaA new species of Tetradesmus (Chlorophyceae, Chlorophyta) isolated from desert soil crust habitats in southwestern North America. Plant Fungal Syst..

[12] Cheng SY, Huang KJ, Wang SK, Wen ZH, Chen PW, Duh CY (2010). Antiviral and anti-inflammatory metabolites from the soft coral sinularia capillosa. J. Nat. Prod..

[13] Yang X, Kang MC, Lee KW, Kang SM, Lee WW, Jeon YJ (2011). Antioxidant activity and cell protective effect of loliolide isolated from *Sargassum ringgoldianum* subsp. coreanum. Algae.

[14] Lee YR, Bae S, Kim JY, Lee J, Cho DH, Kim HS (2019). Monoterpenoid loliolide regulates hair follicle inductivity of human dermal papilla cells by activating the Akt/β-catenin signaling pathway. J. Microbiol. Biotechnol..

[15] Yun JH, Cho DH, Lee B, Kim HS, Chang YK (2018). Application of biosurfactant from *Bacillus subtilis* C9 for controlling cladoceran grazers in algal cultivation systems. Sci. Rep..

[16] Cheng J, Yi X, Wang Y, Huang X, He X (2017). Phenolics from the roots of hairy fig (*Ficus hirta* Vahl.) exert prominent antiinflammatory activity. J. Funct. Foods.

[17] Park Ki-Eui, Kim You Ah, Jung Hyun Ah, Lee Hee-Jung, Ahn Jong-Woong, Seo Y (2004). Three Norisoprenoids from the brown alga *Sargassum thunbergii*. J. Korean Chem. Soc..

[18] Mori K, Khlebnikov V (1993). Carotenoids and degraded carotenoids, VIII -synthesis of (+)-dihydroactinidiolide, (+)- and (−)-actinidiolide, (+)- and (−)-loliolide as well as (+)- and (−)-epiloliolide. Liebigs Ann. der Chemie.

[19] Panich U, Sittithumcharee G, Rathviboon N, Jirawatnotai S (2016). Ultraviolet radiation-induced skin aging: the role of DNA damage and oxidative stress in epidermal stem cell damage mediated skin aging. Stem Cells Int..

[20] Wenk J, Brenneisen P, Meewes C, Wlaschek M, Peters T, Blaudschun R (2001). UV-induced oxidative stress and photoaging. Curr. Probl. Dermatol..

[21] Morrison KC, Hergenrother PJ (2013). Natural products as starting points for the synthesis of complex and diverse compounds. Nat. Prod. Rep..

