# Supplementary material for: Identification of Loliolide with Anti-Aging Properties from Scenedesmus deserticola JD052
Source: J Microbiol Biotechnol. 2023 Jun 2;33(9):1250–6. doi: 10.4014/jmb.2304.04044 (PMC10580889; doi:10.4014/jmb.2304.04044)
Supplement: Supplementary file 1 [file jmb-33-9-1250-supple.pdf]

J. Microbiol. Biotechnol.
https://doi.org/10.4014/jmb.2304.04044

Supplementary Figures

A)

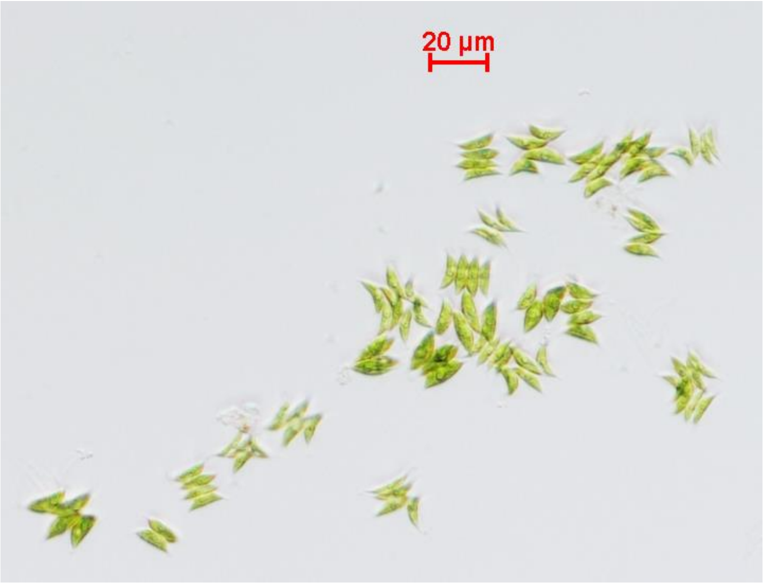

B)

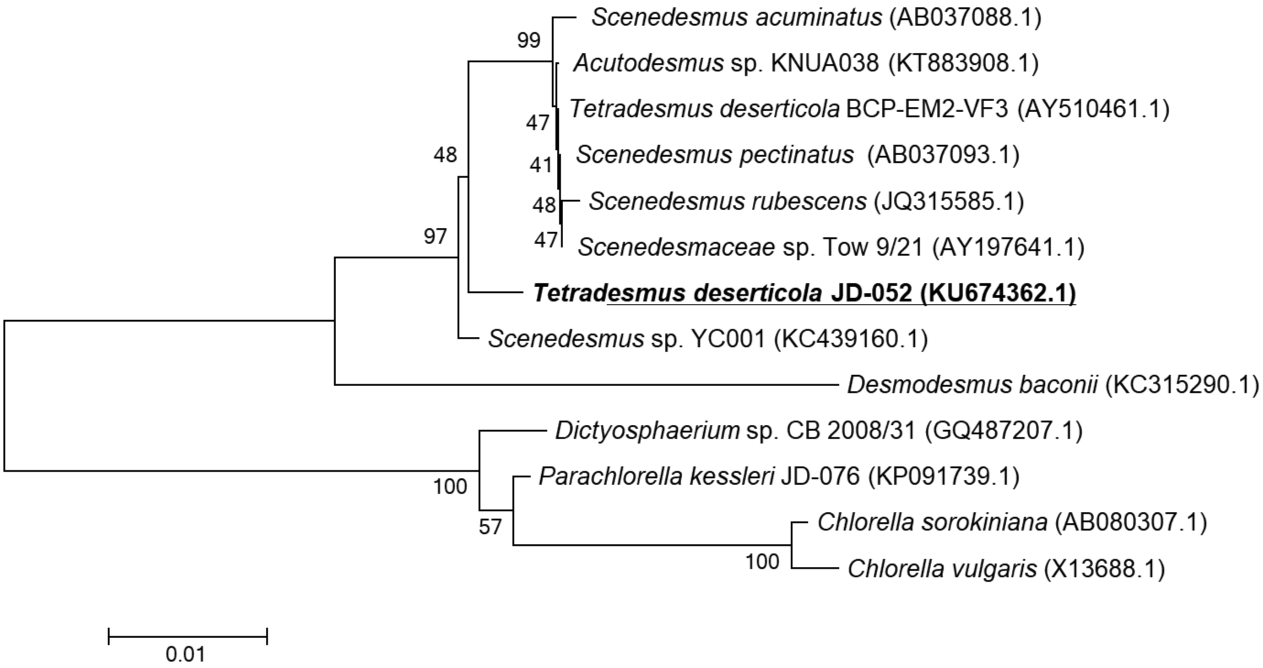

Supplementary Fig. 1. Photomicrography A) and phylogenetic tree B) of *Tetradesmus deserticola* JD-052.

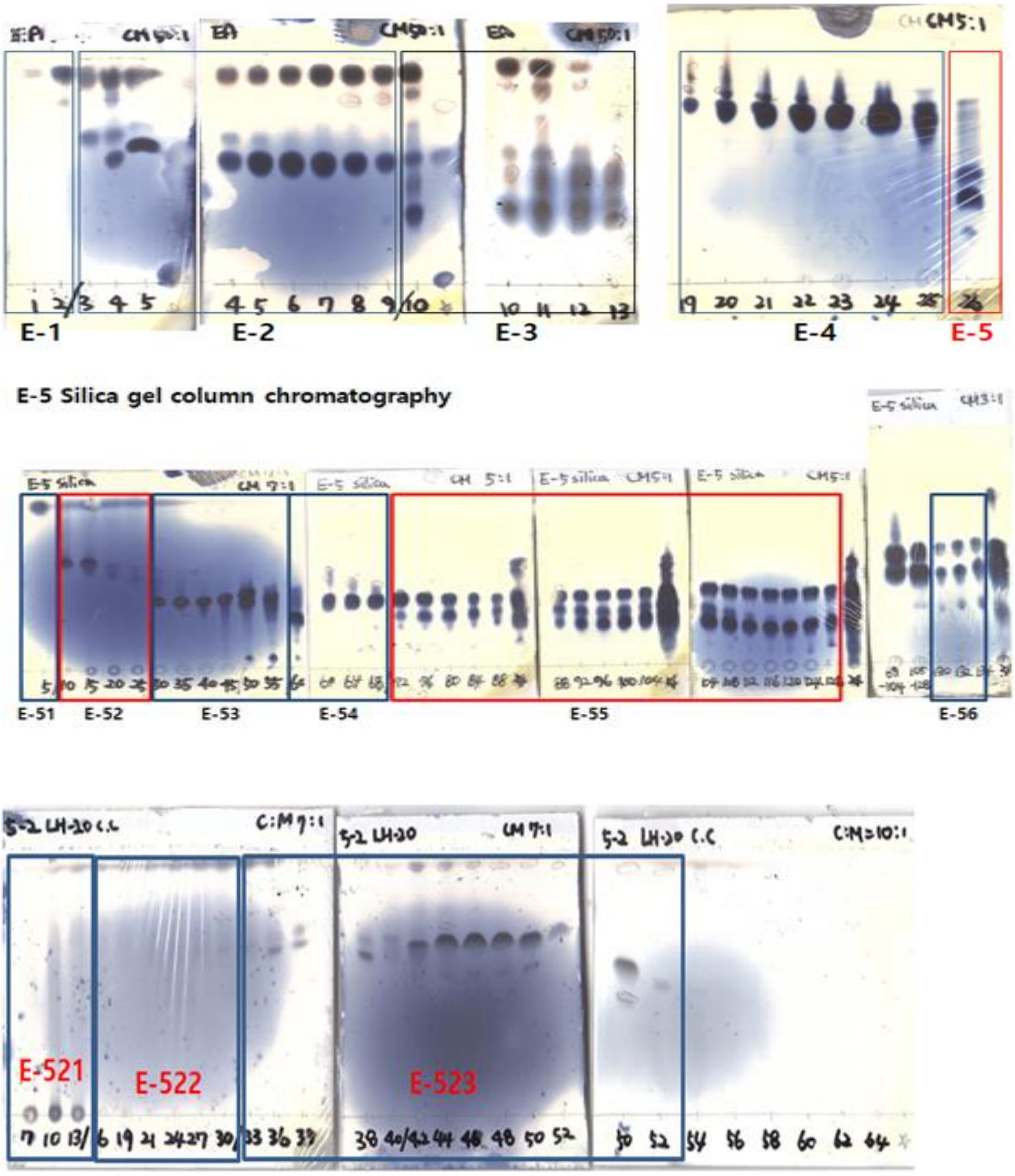

Supplementary Fig. 2. Silica gel TLC of *T. deserticola* JD052 EA extract.

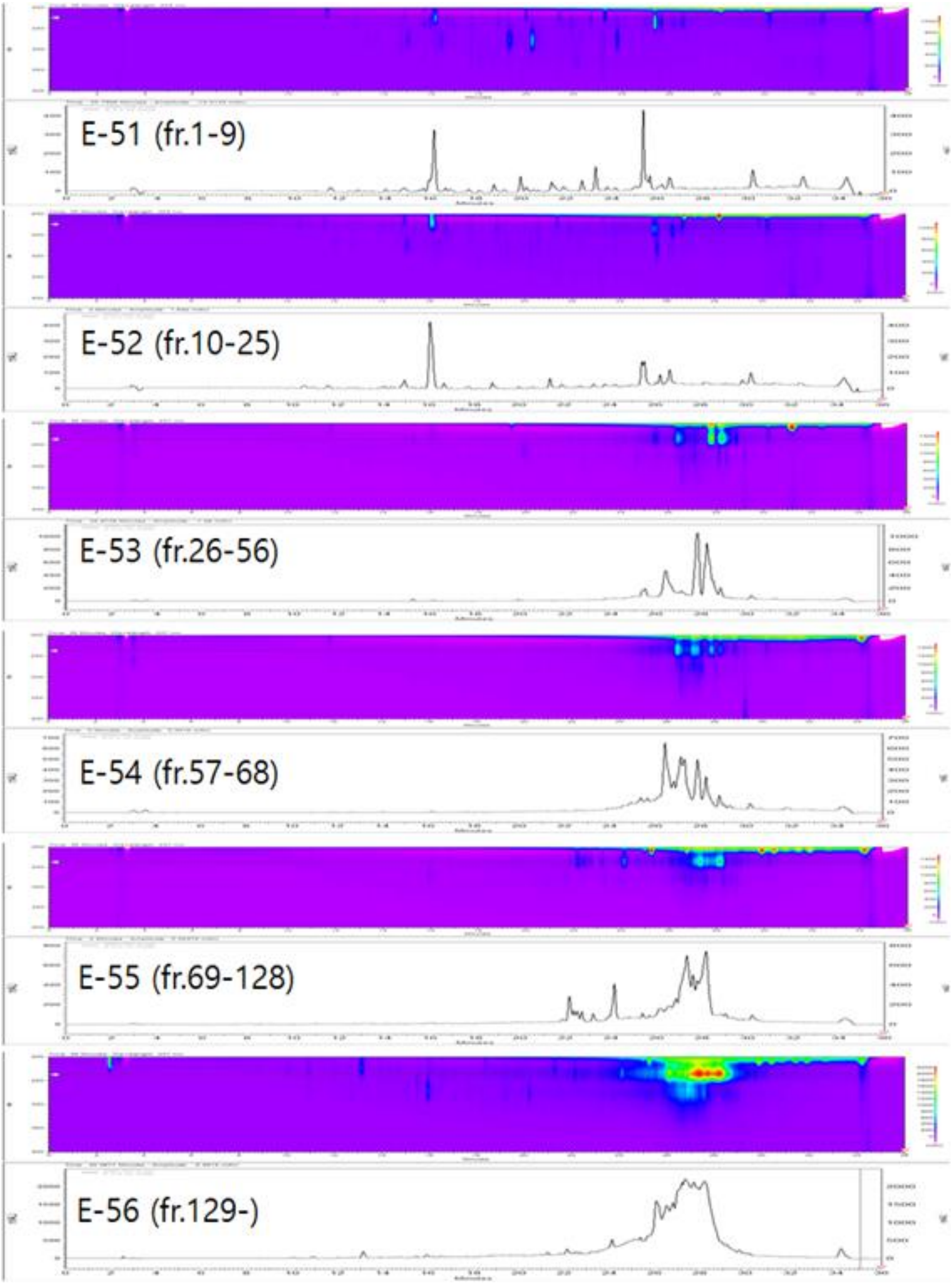
E-51 (fr.1-9)
E-52 (fr.10-25)
E-53 (fr.26-56)
E-54 (fr.57-68)
E-55 (fr.69-128)
E-56 (fr.129-)

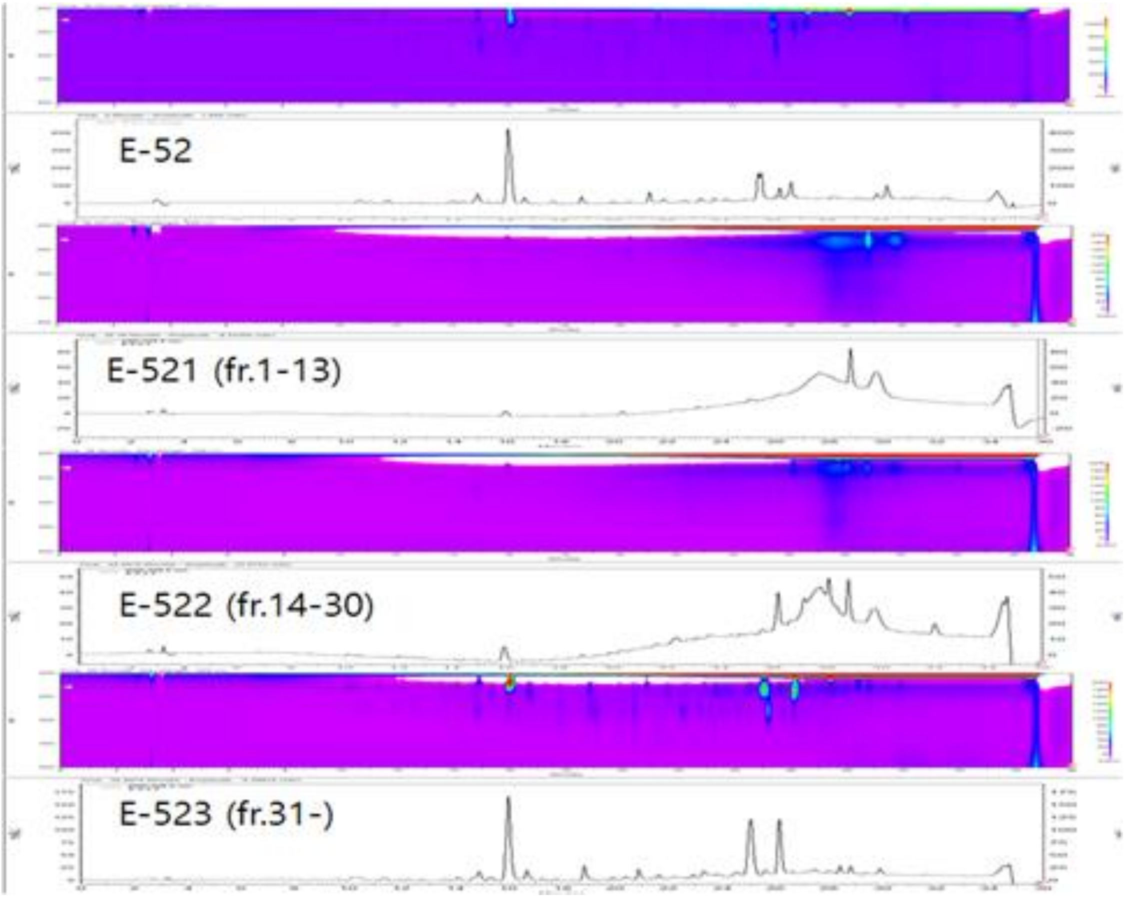

Supplementary Fig. 3. HPLC analysis of *T. deserticola* JD052 EA extract.

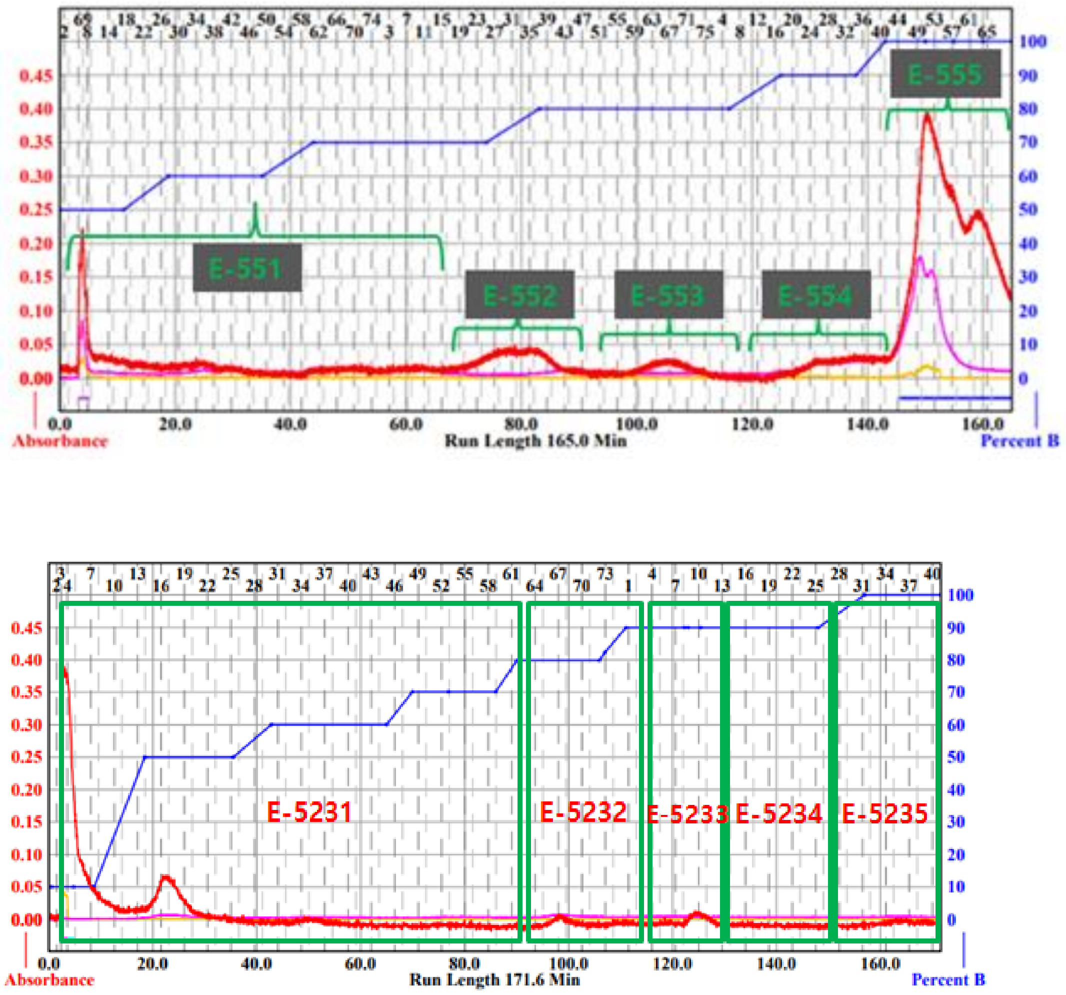

Supplementary Fig. 4. Preparative ODS MPLC analysis of *T. deserticola* JD052 EA extract.

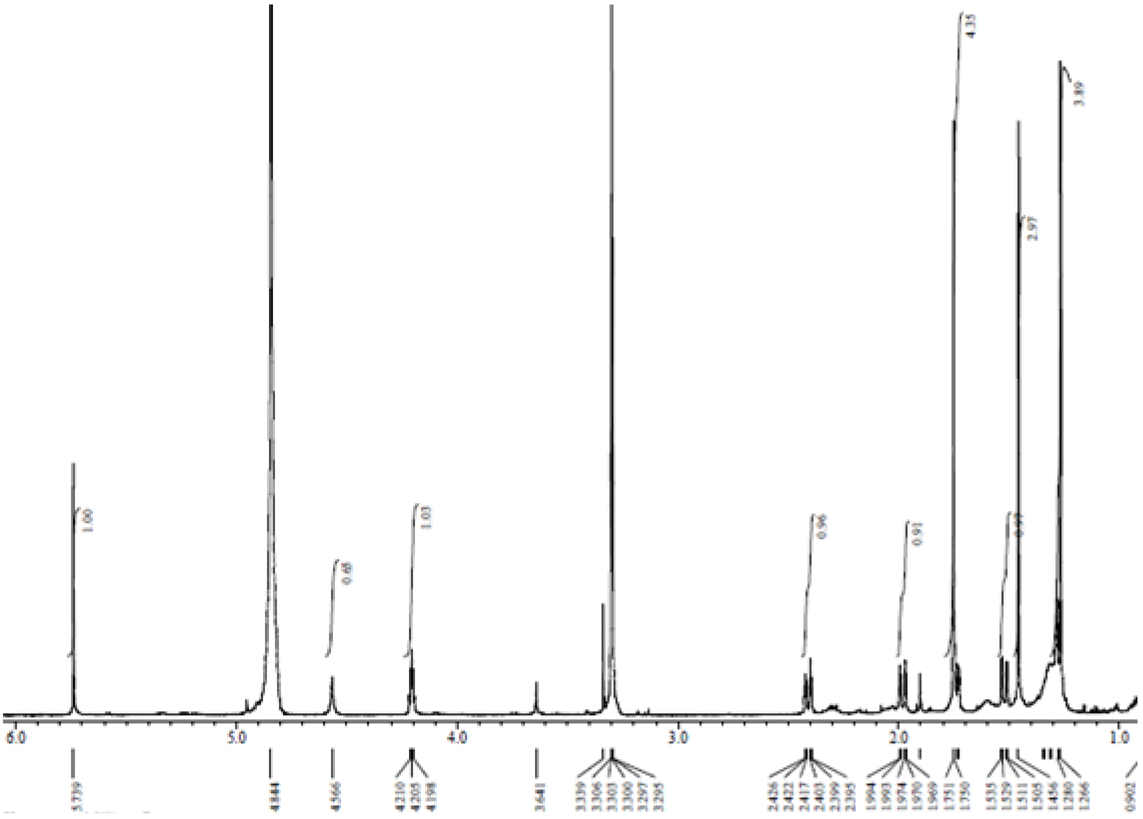

Supplementary Fig. 5. ^{1}H NMR spectrum of E-5231 compound.

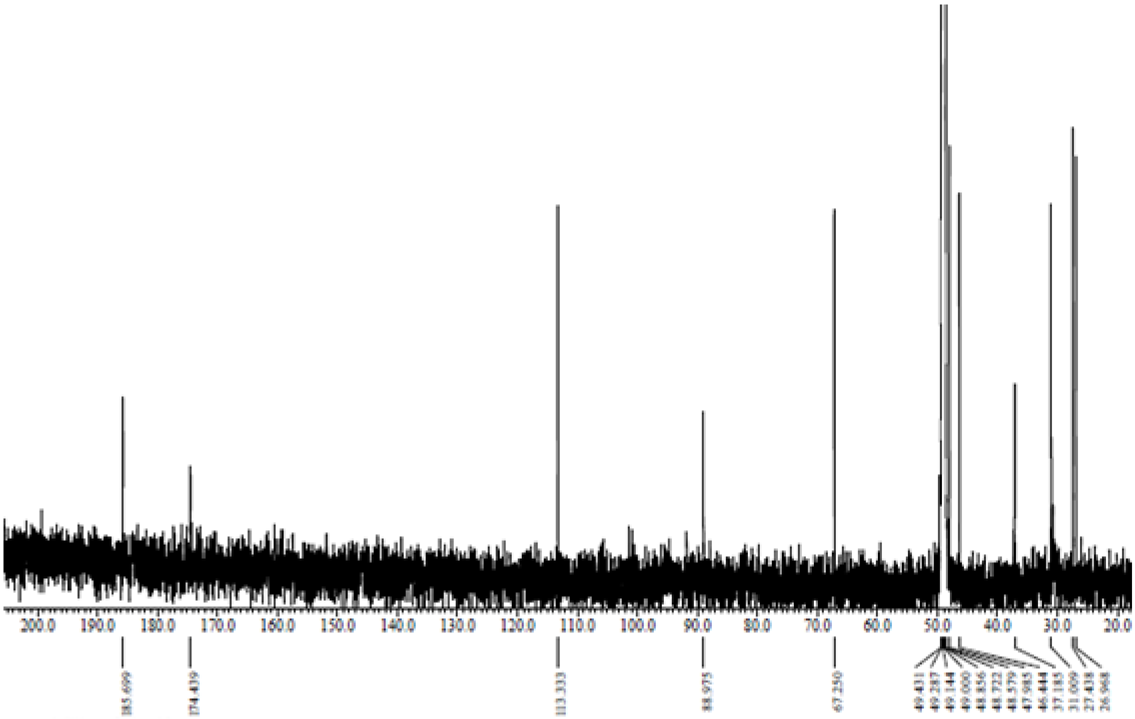

Supplementary Fig. 6. ^{13}C NMR spectrum of E-5231 compound.

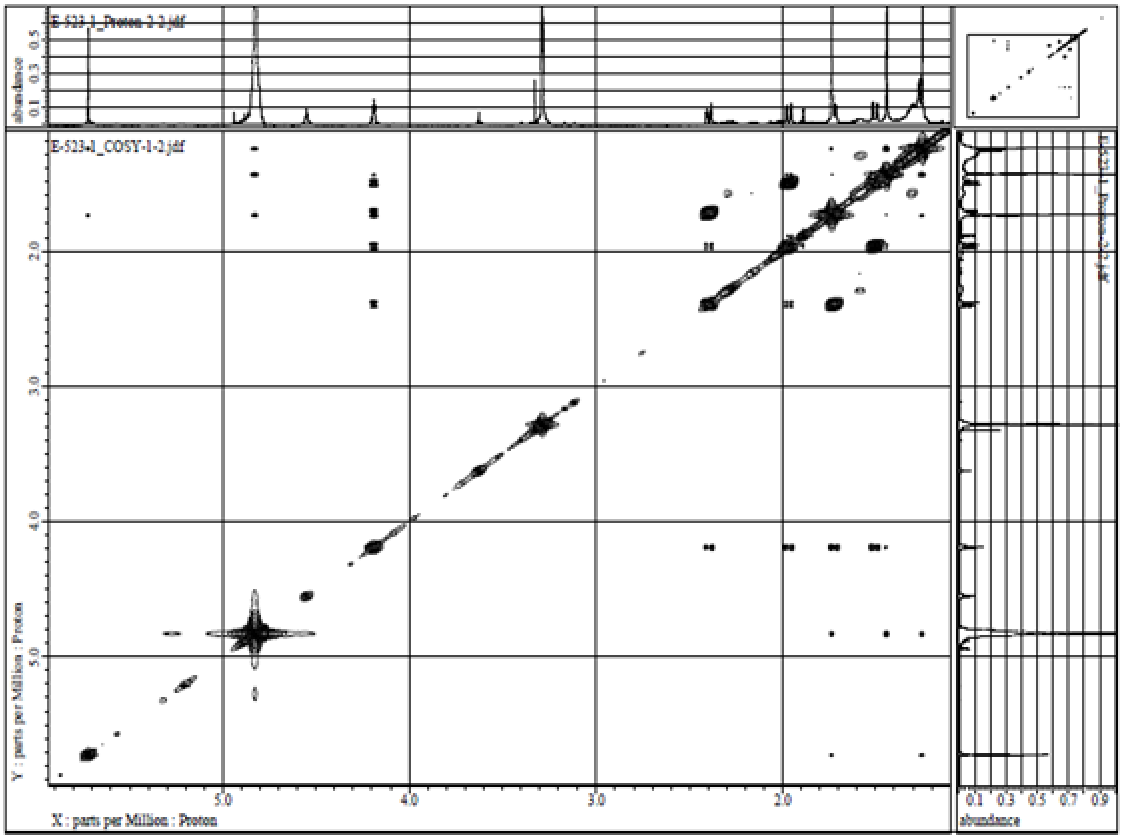

Supplementary Fig. 7. ^{1}H-^{1}H COSY of E-5231 compound

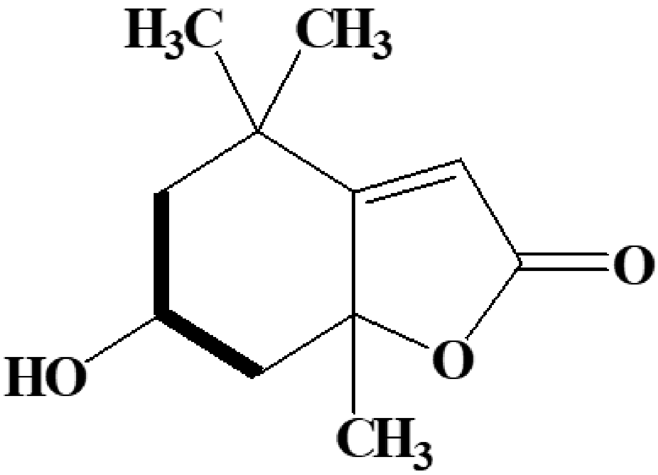

Supplementary Fig. 8. Partial structure by ^{1}H-^{1}H COSY spectrum analysis.

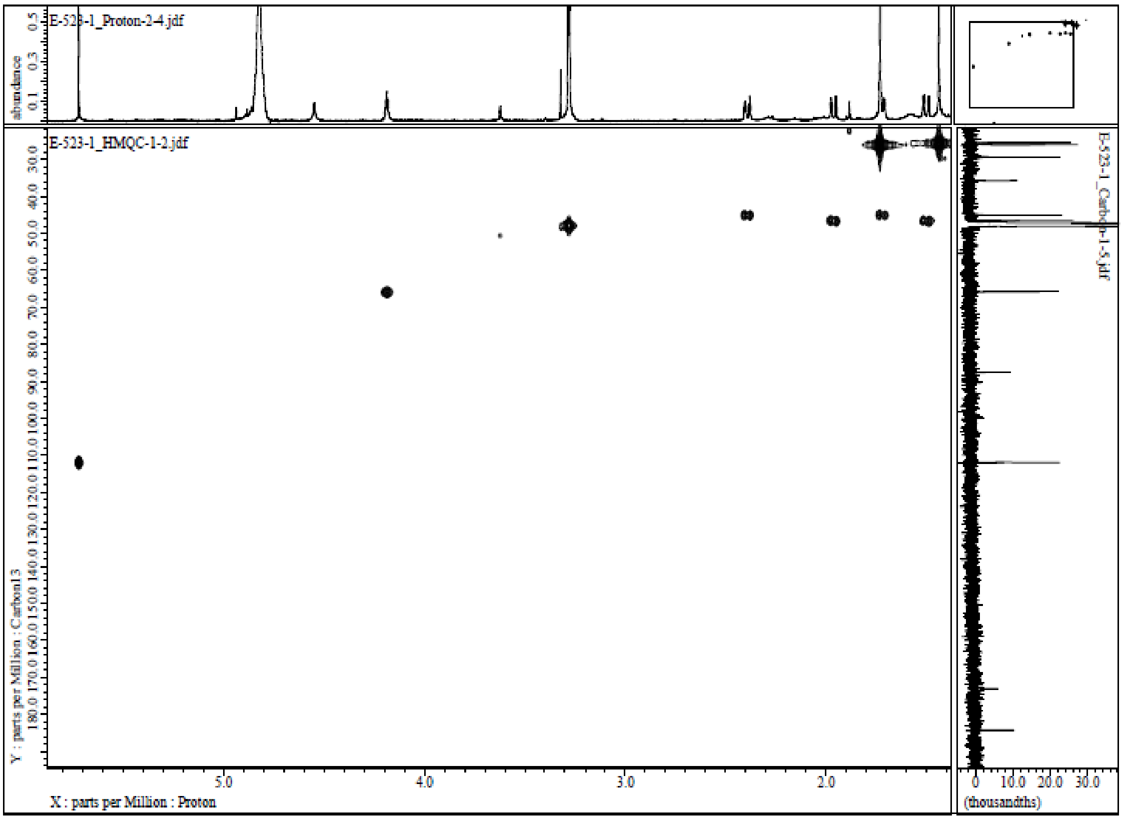

Supplementary Fig. 9. HMQC spectrum by ^{1}H-^{1}H COSY spectrum analysis.

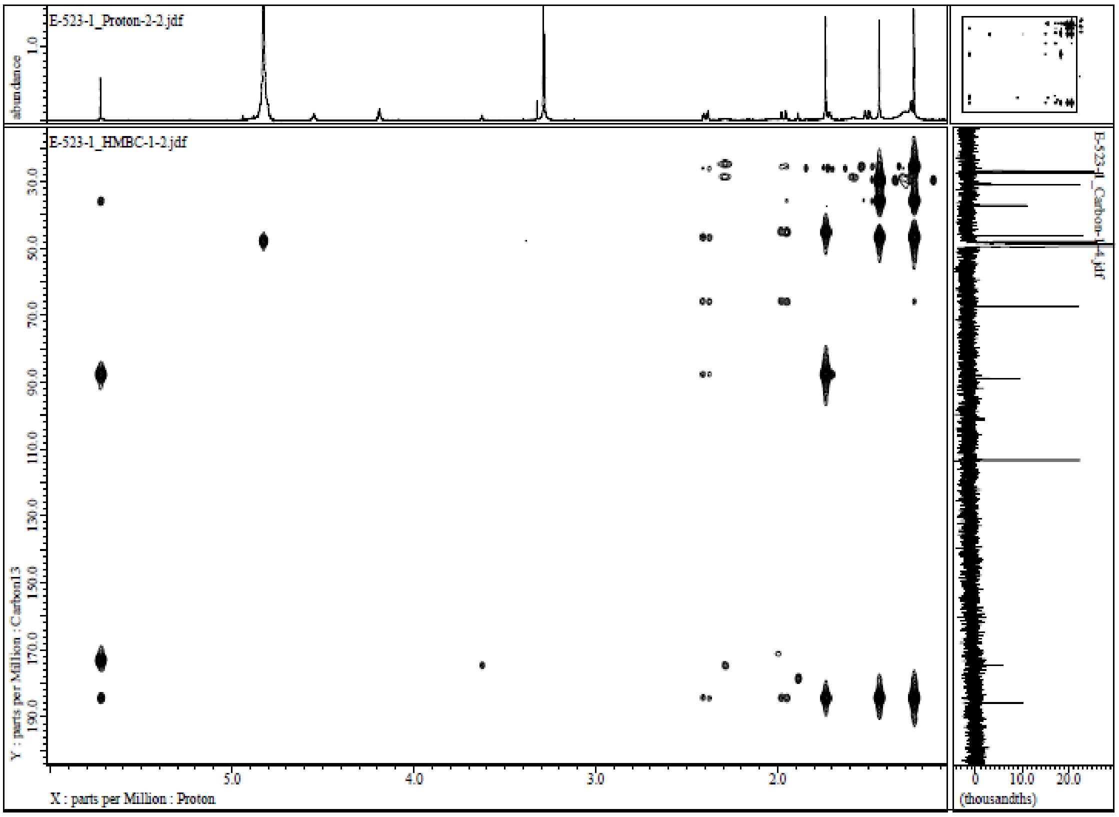

Supplementary Fig. 10. HMBC spectrum by ^{1}H-^{1}H COSY spectrum analysis.
